# NanoTiO_2_ Sunscreen Does Not Prevent Systemic Oxidative Stress Caused by UV Radiation and a Minor Amount of NanoTiO_2_ is Absorbed in Humans

**DOI:** 10.3390/nano9060888

**Published:** 2019-06-17

**Authors:** Daniela Pelclova, Tomas Navratil, Tereza Kacerova, Blanka Zamostna, Zdenka Fenclova, Stepanka Vlckova, Petr Kacer

**Affiliations:** 1 Department of Occupational Medicine, First Faculty of Medicine, Charles University in Prague and General University Hospital in Prague, Na Bojišti 1, 128 00 Prague 2, Czech Republic; zdenka.fenclova@lf1.cuni.cz (Z.F.); Stepanka.Vlckova@vfn.cz (S.V.); 2 J. Heyrovsky Institute of Physical Chemistry of the Czech Academy of Sciences, Dolejskova 3, 182 23 Prague 8, Czech Republic; TNavratil@seznam.cz; 3 Department of Chemistry, University College London, 20 Gordon Street, London WC1H 0AJ, UK; tereza.kacerova.18@ucl.ac.uk; 4 Faculty of Science, Charles University in Prague, Vinicna 5, 128 43 Prague 2, Czech Republic; blanka.zamostna@gmail.com; 5 Czech University of Life Sciences, Kamycka 129, 165 00 Prague 6, Czech Republic; kacerp@af.czu.cz

**Keywords:** sunscreen, UV irradiation, nanoparticles absorption, nanoTiO_2_, oxidative stress, inflammation, nanotoxicology, plasma, urine, exhaled breath condensate (EBC), transmission electron microscopy (TEM), scanning electron microscopy (SEM)

## Abstract

The present pilot study tested the efficiency of nanoTiO_2_ sunscreen to prevent the oxidative stress/inflammation caused by ultraviolet (UV) radiation using biomarkers in subjects’ blood, urine, and exhaled breath condensate (EBC). In addition, the skin absorption of nanoTiO_2_ was studied. Six identical subjects participated in three tests: (A) nanoTiO_2_ sunscreen, (B) UV radiation, and (C) sunscreen + UV. The first samples were collected before the test and the second after sunscreen application and/or UV exposure. On day 4, the third samples were collected, and the sunscreen was washed off, and the fourth samples were collected on day 11. The following biomarkers were measured: malondialdehyde, 4-hydroxy-*trans*-hexenal, 4-hydroxy-*trans*-nonenal, aldehydes C6-C12, 8-*iso*-Prostaglandin F2α, o-tyrosine, 3-chlorotyrosine, 3-nitrotyrosine, 8-hydroxy-2-deoxyguanosine, 8-hydroxyguanosine, 5-hydroxymethyl uracil, and leukotrienes, using liquid chromatography-electrospray ionisation-tandem mass spectrometry. Titania was measured using inductively coupled plasma mass spectrometry and TiO_2_ nanoparticles by transmission and scanning electron microscopy. Sunscreen alone did not elevate the markers, but UV increased the biomarkers in the plasma, urine, and EBC. The sunscreen prevented skin redness, however it did not inhibit the elevation of oxidative stress/inflammatory markers. Titania and nanoTiO_2_ particles were found in the plasma and urine (but not in the EBC) in all sunscreen users, suggesting their skin absorption.

## 1. Introduction

Skin cancer prevention is important and sunscreen use has been recommended as a cost-effective preventative, especially in countries with a high-sunlight environment [1,2]. It has been estimated that a majority of cancers could potentially be reduced by regular sunscreen use. It included around 9.3% of squamous cell skin carcinomas and 14% melanomas that may be prevented if UV exposure was reduced. On the other hand, the effect of the sunscreen concerning the prevention of basal cells carcinomas’ incidence was not seen [1]. The studies showing effect mostly used sunscreens with organic filters, not sunscreens with nanoparticles [2]. However, some studies have reported potentially unfavourable effects of sunscreens, since they can extend the duration of sunbathing and increase the risk of skin malignancies [3,4,5,6]. Other studies point to a high reactivity of nanoparticles and their potential to produce reactive oxygen species (ROS), alter the skin structure [7,8], and/or penetrate the skin [9].

Ultraviolet (UV) radiation, encompassing UVA (wavelengths 320–400 nm), UVB (280–320 nm), and UVC (200–280 nm), including UV-emitting tanning devices, may cause each of the three main types of skin cancer: basal cell carcinoma, squamous cell carcinoma, and melanoma [10].

While UVA and UVB rays are transmitted through the atmosphere, all UVC and some UVB rays are absorbed by the Earth’s ozone layer. Thus, most of the UV rays we encounter are UVA (95%).

UVA penetrates deeply into the dermal layer of the skin, promoting skin aging, wrinkles, and in large doses, cancer [11,12,13]. Only 10%–30% of UVB reaches the surface, but its biological effects are larger. UVB radiation only enters the epidermis and triggers erythema, pigmentation, and optical aging, however, it can cause chemical changes in DNA, increasing the risk of skin cancer [12]. On the other hand, UVB is also known to play positive roles, including supporting the synthesis of vitamin D in the human body [13].

Similar to the sun, tanning beds typically emit 95% UVA and 5% UVB [11].

There are several complex mechanisms by which non-melanoma and melanoma skin cancers can be attributed to DNA damage caused by UV radiation exposure (photocarcinogenesis), involving the interplay between various biochemical processes [14]. UV can generate ROS, such as hydroxyl radicals (OH·), hydrogen peroxide (H_2_O_2_), and superoxide anions (O_2_^−^), leading to oxidative stress, which is considered a key mechanism of cell and DNA damage. It was recently shown that the use of antioxidants, such as ascorbic acid and rutin, appears beneficial following UV irradiation [15].

The formation of a marker of the peroxidation of lipids, such as 8-*iso*-prostaglandin F2α (8-isoprostane) by free-radical lipid peroxidation of arachidonic acid, has been detected in human skin in response to UVB radiation [16]. Another lipid peroxidation product, malondialdehyde (MDA), can accumulate in human skin and MDA-protein adducts have also been found in skin cancer. UV radiation induces mutation of nucleotides that are highly susceptible to these free radical injuries [13]. For instance, guanine oxidation leads to the formation of 8-hydroxy-2-deoxyguanine (8-OHdG), and changes in subsequent base pairing can cause mutations, as has been found in skin cancer.

Sunscreens should be effective in blocking both the UVA and UVB spectrum. Unique properties of nanoparticles have led to an increase in their use in various applications, including medicine [2,17], however, there are also concerns regarding the safety of nanoTiO_2_, not solely in sunscreens.

One of the most frequently used nanoparticles is titania (TiO_2_), which has the ability to reflect, scatter, and absorb UV radiation [18], however this results in photocatalysis, releasing ROS capable of altering DNA [11]. Additionally, oxidative damage and genotoxicity without UV radiation have been found in vivo [19,20].

Based on animal studies, TiO_2_ was reclassified in 2010 by the International Agency for Research on Cancer as a group 2B carcinogen, i.e., possibly carcinogenic to humans [21]. In the experimental studies, nanoTiO_2_ toxicity was higher than that of bulk TiO_2_ due to the higher reactivity of the nanoparticles, since they have a highly active surface area (hundreds m^2^/g). This is due to three main mechanisms: (1) ROS production following the induction of electron-hole pairs, (2) damage of the cell membranes due to lipid peroxidation by the attachment of nanoparticles to cells via electrostatic forces as a result of their large specific surface area, and (3) TiO_2_ nanoparticle attachment to intracellular organelles and macromolecules following cell membrane damage [20].

TiO_2_ can be found in three crystalline structures that differ in their potential permeation, i.e., anatase, rutile, and brookite, in addition to an amorphous phase. Rutile is more desirable for use in sunscreens, since it is less photoreactive [11]. Both the anatase and rutile forms, including anatase/rutile combinations, can generate ROS [22] and perturb the structure of the stratum corneum [8].

There are concerns regarding the potential penetration of nanoTiO_2_ particles from sunscreens into viable skin, and subsequent systemic absorption, however the conclusions are ambiguous, since these data are very limited [23,24].

One earlier study confirmed that nanoTiO_2_ absorption occurred from sunscreens that were used twice daily for 9–31 days in 13 patients scheduled for skin surgery [25]. In subsequent histological examination, TiO_2_ was detected in both the epidermis and dermis. In another study in two volunteers, TiO_2_ nanoparticles were found in viable cells of the epidermis following sunscreen application six times a day for seven consecutive days. However, these studies did not ascertain whether the particles were translocated into the systemic circulation [18].

To date, several molecular epidemiological studies in workers exposed to different nanoparticles during handling engineered nanomaterials suggest health impairment [26]. Elevated pro-inflammatory markers, antioxidant enzymes, cardiovascular markers [27,28], pro-inflammatory leukotrienes (LTs), and tumour necrosis factor [29,30,31] in the circulation, markers of oxidative stress in the exhaled breath condensate (EBC) and/or circulation, and impaired lung functions have been observed [32,33,34,35,36,37].

EBC is a liquid, collected during tidal breathing, which is composed mainly of water (99.9%) and contains only a small proportion of water-soluble and insoluble compounds, presumably originating from the airway lining fluid in the form of aerosolised particles generated during the re-opening of distal airways [38].

Unlike the non-invasive marker of eosinophilic airway inflammation, fractional exhaled nitric oxide (FeNO) [39], EBC is not considered a standardised biological specimen. Its analysis is used in research but not yet in clinical practice, since the percentage of condensed liquid of the exhaled volume is not constant for each collection process. Nevertheless, it enables the non-invasive collection and detection of a broad spectrum of biomarkers and particles from the airways and lungs [38].

Oxidative alteration of lipids occurs in vivo during aging and in certain health disorders. It includes stable 8-isoprostane and unstable indicators of oxidative stress in cells, such as lipid peroxides forming reactive compounds, i.e., MDA, 4-hydroxy-*trans*-hexenal (HHE), and 4-hydroxy-*trans*-nonenal (HNE), which can covalently bind to DNA and proteins, and are therefore considered genotoxic and cytotoxic [40]. The stable products formed from peroxynitrite (ONOO-) and hypochlorous acid (HClO) with tyrosine residues in proteins are 3-nitrotyrosine (3-NOTyr) and 3-chlorotyrosine (3-ClTyr) respectively, both of which are related to neutrophilic inflammation and have been found in patients with interstitial respiratory disorders, in addition to o-tyrosine (o-Tyr) [40]. Finally, 8-OHdG and 8-hydroxyguanosine (8-OHG), formed by the oxidation of guanine in DNA and 5-hydroxymethyl uracil (5-OHMeU) in RNA respectively, reflect oxidative damage to nucleic acids [40].

Several biomarkers of inflammation have been successfully measured in EBC using highly sensitive techniques, such as liquid chromatography mass spectrometry (LC/MS). LTB4 activates leukocytes and induces chronic neutrophilic inflammation. Cysteinyl LTs (LTC4, LTD4, and LTE4) increase vascular permeability and induce airway smooth-muscle contraction [41]. Proinflammatory LTs have been found in workers exposed to nanoTiO_2_ for an entire shift, but also in office workers with only an approximate 15-min of daily exposure [29,30,31,42,43].

Although the use of sunscreen containing nanoTiO_2_ is extensive, a limited number of human studies have been conducted to assess the response triggered by the nanoparticles and the uptake of nanoTiO_2_ by human skin. In the present pilot study, biological fluids taken non-invasively, such as EBC, blood, and urine, were used to study the effects of sunscreen in humans. We aimed to test:

(1) potentially deleterious effects of commercially available nanoTiO_2_ sunscreen by measuring markers of oxidative stress and/or inflammation in plasma, urine, and EBC;
(2) beneficial preventive effects of commercial nanoTiO_2_ sunscreen in limiting/blocking oxidative stress and inflammatory markers in plasma, urine, and EBC, triggered by UV radiation from a commercial tanning bed;
(3) potential absorption of titania and TiO_2_ nanoparticles by their presence in biological fluids—plasma and urine—prior to, during, and one week after sunscreen use;
(4) potential inhalational absorption of titania and TiO_2_ nanoparticles (as powder contamination from the dry sunscreen on skin) by analysing the EBC in the collected samples;
(5) spirometry and FeNO to evaluate potential respiratory disorders.

## 2. Materials and Methods

### 2.1. Subjects and Biological Sample Collection

Six Caucasian volunteers (3 males and 3 females), all non-smokers with a mean age of 48.0 ± 8.3 years old, participated in the present study, which was approved by the Ethical Committee of Charles University according to the Helsinki criteria. All participants were informed of the study aim and signed an informed consent form prior to the beginning of the study. All participants filled in a standardised questionnaire that included potential sun exposure and food consumption of potential sources of TiO_2_ (yoghurts, chewing gums, etc.), and their blood, urine, and EBC were subsequently collected. FeNO examination and spirometry were then carried out.

These identical subjects participated in three test variants: study (A) commercial nanoTiO_2_ sunscreen continuously for 3 days with 2 applications/day on approximately 80% of the body surface; study (B) 2× UV tanning bed; study (C) both sunscreen and 2× UV. The schedule for the three test variants is shown in Table 1. All studies were carried out in Prague, the interval between each of the three studies was at least 3 months.

The first sample (blood plasma, urine, EBC) was collected at the beginning of the test, and the second sample was taken following sunscreen application and/or the first UV exposure. On day 3, a second UV exposure occurred in variants B and C.

The sunscreen was applied (in variants A and C) to approximately 80% of the body surface (excluding head, palms, cubital areas, soles, and bikini area) twice daily for 3 days and was not washed off. The subjects wore white shirts and white trousers that remained unchanged until day 4. After the third sample was collected, the used sunscreen (in variants A and C) was thoroughly washed off. The fourth sample was collected on day 11, i.e., one week after the end of sunscreen exposure, if used.

In all test variants, 4 samples of blood plasma, urine, and EBC were collected at the same time intervals.

### 2.2. Sunscreen

#### 2.2.1. Label Information for the Commercial Sunscreen

The sunscreen used in studies A and C had a high skin protection factor (SPF) of 50, UBV filter 50, described as shielding against sunburn, in addition to a UVA filter of 98% and vitamin E, described as safeguards against sun-induced damage and aging. The sunscreen was labelled as oil-free, alcohol-free, free from paraffin, parabens, polyethylene glycol bonds and para-aminobenzoic acid esters, acrylamide, and colour additives, and also suitable for children and subjects with very sensitive and sun-intolerant skin. The pH was 5.5, with a total content of 150 g.

Ingredients were marked as follows: aqua, dibutyl adipate, octocrylene, titanium dioxide/nano), C12-15 alkyl benzoate, glycerine, dimethicone, butyl methoxydibenzoylmethane, diethylamino hydroxybenzoyl hexyl benzoate, VP/eicosene copolymer, bis-ethylhexyloxyphenol methoxyphenyl triazine, microcrystalline cellulose, panthenol, tocopheryl acetate, diethylhexyl butamidotriazone, ethylhexylglycerin, sodium cetearyl sulphate, silica, benzyl alcohol, parfum, xanthan gum, sorbic acid, disodium ethylenediaminetetraacetic acid (EDTA), lauryl glucoside, polyglyceryl-2 dipolyhydroxystearate, cellulose gum, glyceryl stearate, cetearyl alcohol, cetyl palmitate, cocoglycerides, phenoxyethanol, dehydroacetic acid, inulin, and lecithin.

The recommended dosage was 2 mg/cm^2^ skin, i.e., approximately 6 teaspoons (30 g)/adult, 20 min prior to UV exposure and to be re-applied frequently to maintain protection at a sufficient level, especially after perspiring, swimming, or towelling (typically twice a day).

Neither the crystalline structure of nanoTiO_2_ nor the possible coating of the nanoparticles (with Si or Al) were specified. The TiO_2_ content was not described, however, it should not exceed the limit of 25% *w*/*w* in EU products [44].

#### 2.2.2. X-ray Powder Diffraction (XRD)

Crystallographic measurements of the commercially available sunscreen were carried out by a four circle charge-coupled device (CCD) diffractometer (Gemini of Oxford Diffraction, Ltd., Oxford, UK) using graphite monochromated Mo Kα radiation (λ = 0.71073 Å). The crystal structure was solved by the charge-flipping method using the Superflip program and refined using the Jana2006 program package by the full-matrix least-squares technique. The structure plots were prepared using ORTEP III. Particle size was counted as one point, always from the most intense line of the sample being measured.

#### 2.2.3. Microscopy Techniques

Scanning electron microscopy (SEM) of the sunscreen was performed using a scanning electron microscope (Hitachi S-4700, Hitachi, Tokyo, Japan) with a cold cathode (resolution up to 1.5 nm (15 kV)) and equipped with two secondary electron detectors and one detector for scattered electrons. Acquisition of high-quality images and interpretation was carried out by multiparameter optimisation, which optimised the cathode emission current, excitation condenser aperture size, working distance, accelerating voltage, specimen geometry, and detectors. The microscope itself offered the possibility to set the accelerating voltage from 0.5 to 30 kV; the resolution was 1.5 nm, and the magnification was 20–500,000×.

### 2.3. UV Exposure

Two sunbed sessions in the same commercial solarium provided radiation under the limit of 0.3 W/m^2^, i.e., the amount of UV exposure that the EU’s Scientific Committee on Consumer Products has set as the limit for effective irradiance (EN 60335-2-27). Under this standard, a commercial sunbed session shall have a maximum UV output that corresponds to the mid-day Mediterranean sun (UV index of 12) to avoid any risk of burns [44]. The time of exposure (2 × 8 min) and interval between the two exposures (48 h) followed the recommended limits. Body insolation was in the lying position on both sides in a bikini suit, similar to the real situation, in addition, protective glasses were used.

### 2.4. Exhaled Breath Condensate (EBC) Sample Collection and Analysis

EBC samples were collected using an Ecoscreen Turbo (DECCS, Jaeger, Hochberg, Germany). All subjects wore a nose-clip to exclude nasal contamination and breathed tidally for approximately 15 min through a mouthpiece connected to the condenser (−20 °C). A minimum exhaled air volume was measured by an EcoVent device (Jaeger, Hochberg, Germany) to reach a sufficient volume (at least 120 litres). All samples were immediately spiked with deuterium-labelled standards, frozen, and stored at −80 °C until processing. The pH of EBC was measured, and to exclude contamination of EBC by saliva, the alpha-amylase concentration was determined [45].

### 2.5. Analysis of Markers of Oxidative Stress and Inflammation

A panel of markers of oxidative stress originating from the free radical oxidation of lipids, nucleic acids, and proteins, and a panel of low-molecular inflammatory biomarker LTs was analysed in plasma, urine, and EBC. These markers included MDA, HHE, HNE, aldehydes C6-C12, 8-isoprostane, o-Tyr, 3-ClTyr, 3-NOTyr, 8-OHdG, 8-OHG, 5-OHMeU, and LTs. The analysis was performed following solid-phase extraction (SPE) using high-performance liquid chromatography-electrospray ionization-tandem mass spectrometry (HPLC-ESI-MS/MS), using a quaternary pump, Accela 600, and Accela autosampler coupled to a triple quadrupole mass spectrometer TSQ Vantage equipped with heated electrospray ionisation (HESI) (Thermo Fisher Scientific, Thermo Waltham, MA, USA) [46,47,48,49]. Post-exposure markers in samples 2, 3, and 4 were compared with pre-exposure sample 1. All samples were blinded to the personnel involved.

### 2.6. TiO_2_ Particle Analysis in Plasma, Urine, and EBC

#### 2.6.1. Quantitative Determination of TiO_2_ by Inductively Coupled Plasma Mass Spectrometry (ICP-MS)

Quantitative analysis of TiO_2_ in plasma, spot urine, and EBC samples was performed using the ICP-MS technique. An Agilent 7900 ICP-MS Ultra HMI (Agilent, Headquarters, Santa Clara, USA), equipped with the MassHunter software and an autosampler ASX-520 (Agilent, Headquarters, Santa Clara, USA), was used. Prior to measurement, the liquid samples were evaporated to dryness and mineralised with a mixture of HF and HNO_3_ (1:3, *v*/*v*) in a UniClever microwave decomposition unit (Plazmatronika-Service, Wroclaw, Poland). The method was validated and used for quantitative measurements. The limit of detection (LOD) was 1.2 ± 0.2 ng/mL, the limit of quantitation (LOQ) was 4.0 ± 0.2 ng/mL, and the standard error was 3.0%.

#### 2.6.2. Transmission Electron Microscopy (TEM) Particle Analysis

TEM of the plasma, spot urine, and EBC samples was performed using the laboratory JEM-1011 (Jeol, Tokyo, Japan). A 100-µL aliquot of each biological sample was frozen and lyophilised. The residue was dissolved in 10 µL acetone, inserted into the TEM target, and the measurement was performed. Micrographs were obtained, and statistical analysis was carried out. The average particle size and particle size distribution (PSD) were calculated based on 10,000 identified particles. In cases where the number of identified particles did not reach this value, the calculation was performed with all positively identified particles larger than 1 nm.

### 2.7. FeNO Measurement

FeNO was measured by a portable Hypair FeNO analyser (Medisoft, Sorinnes, Belgium). Based on American Thoracic Society and European Respiratory Society (ATS/ERS) recommendations, a FeNO result greater than 50 ppb was considered elevated [39].

### 2.8. Spirometry

Spirometry was carried out by a SpiroPro (Jaeger, Hochberg, Germany). The measurement included forced vital capacity (FVC), inspiratory vital capacity (VCIN), peak expiratory flow (PEF), and forced expiratory volume in 1 s (FEV1). These parameters were considered normal if they reached at least 80% of the predicted values (i.e., comparison with populations with similar characteristics, such as age, gender, height, and weight).

### 2.9. Statistical Analysis

Basic descriptive statistics (mean, median, confidence interval, standard deviation, skewness, and kurtosis) were computed and subsequently tested for normality using the Kolmogorov–Smirnov test. For comparison of frequency counts of demographic categorical variables, Fischer’s exact test was used. Differences in interval variables (e.g., spirometry parameters, markers of inflammation in EBC, and FeNO) were tested using the Mann–Whitney U test). A paired sample *t*-test (or the Wilcoxon signed-rank test) was used to compare pre-exposure values of the markers (in sample 1) with the values of samples 2, 3, and 4. The bivariate relationship between the variables under study was assessed using Spearman’s correlation coefficient. Statistical significance was set at *p* < 0.05. All analyses were conducted using SPSS version 22.0 (SPSS, Inc., Chicago, IL, USA).

## 3. Results

### 3.1. Subjects

All subjects belonged to type III of the Fitzpatrick skin type scale, with no increased sensitivity to sunburn. No subject had skin type I, i.e., blonde to red hair with pale skin with freckles.

Two subjects reported allergic rhinitis without treatment, one woman was being treated with thyroxin for hypothyroidism and with local corticosteroids for bronchial asthma. No subject had chronic bronchitis or dyspnoea.

### 3.2. Sunscreen Use and Analysis

The total average cream consumption in both test variants using the sunscreen was 138.5 ± 9.9 g/testing period. The absolute consumption was higher in males than in females (144.3 g versus 132.7 g respectively), however, the consumption per body surface in males (85.0 ± 4.8 g/m^2^) did not significantly differ from that in females (95.3 ± 6.1 g/m^2^) (*p* = 0.084).

**Figure 1:**
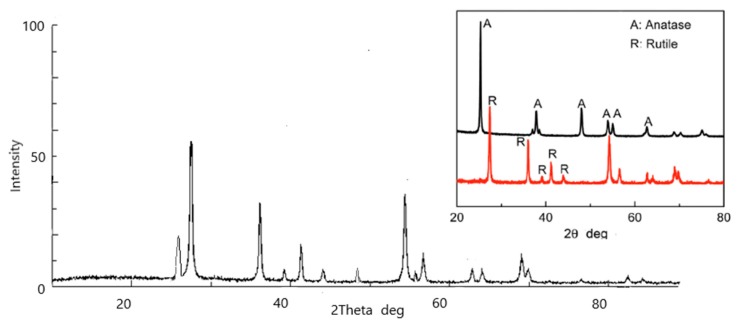
X-ray diffractogram of TiO2 from the commercial sunscreen with an average particle size of 43 nm (determined by XRD); inner image – XRD of rutile standard (R) and anatase standard (A).

X-ray diffraction (XRD) analysis of the commercial sunscreen confirmed the presence of TiO_2_ nanoparticles with an average particle size of approximately 43 nm. The ratio of rutile to anatase was approximately 80:20. In addition, a small portion of SiO_2_ (roughly 2%) was determined by XRD. ZnO was not detected. A detailed physico-chemical characteristics and measurement of TiO_2_ content will be published separately. The X-ray diffractogram of the sunscreen after extraction of the cream matrix and sampling is shown in Figure 1.

**Figure 2:**
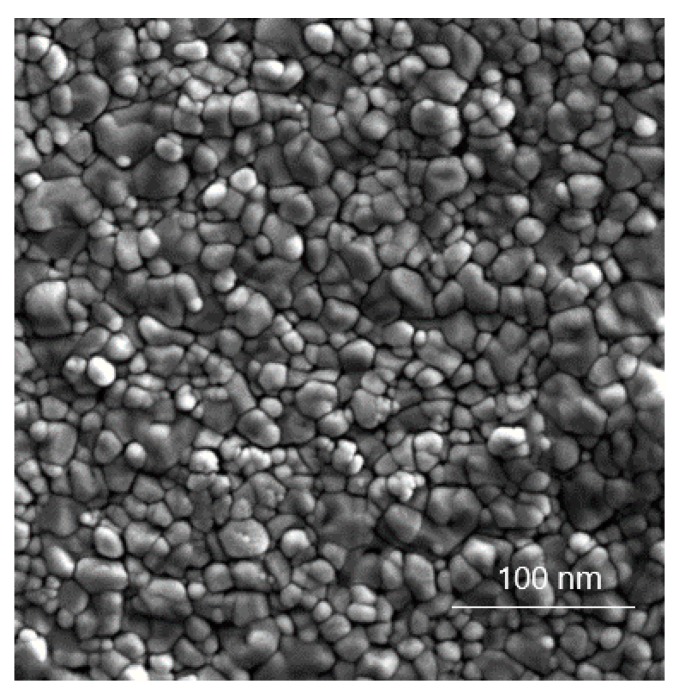
Scanning electron microscopy (SEM) micrograph of particles (after separation of the sunscreen base by extraction) present in the commercially available nanoTiO_2_ sunscreen. Magnification bar = 100 nm.

SEM analysis of the sunscreen is shown in Figure 2. The micrograph of TiO_2_ particles was taken after the separation of the cream matrix by extraction. The particle size distribution was determined as a monomodal distribution in a range of approximately 20–60 nm, with a global maximum of 32 nm. In agreement with XRD, the calculated average particle size was determined to be 43 nm.

### 3.3. UV Exposure

The duration of UV exposure on the tanning bed was 8 min × 2, i.e., a total of 16 min over two sessions.

A very mild erythema followed by tanning occurred after study variant B (UV only), which was not seen in study C (sunscreen + UV). No subject experienced skin burning after UV exposure on the tanning beds in any study.

### 3.4. EBC Alpha-Amylase Concentration and pH

The amylase concentration in all samples was less than 0.01% of the alpha-amylase concentration in saliva. There was no significant difference in the pH of EBC samples (pH range 5.8–6.1).

### 3.5. Markers of Oxidative Stress and Inflammation in Plasma, Urine, and EBC

Study A with solely sunscreen use did not find elevation of any markers of oxidative stress and/or inflammation in plasma samples 2, 3, or 4, as compared with sample 1. In urine, only LTC4 was higher in sample 3 (*p* < 0.05).

A few biomarkers were increased in EBC, i.e., 8-isoprostane in samples 2 and 4 (*p* < 0.05), LTD4 (*p* < 0.01) and LTE4 (*p* < 0.05) in sample 2, 3-ClTyr in sample 3 (*p* < 0.05), and LTC4 (*p* < 0.05) and LTD4 (*p* < 0.001) in sample 4. The data are not shown.

**Figure 3:**
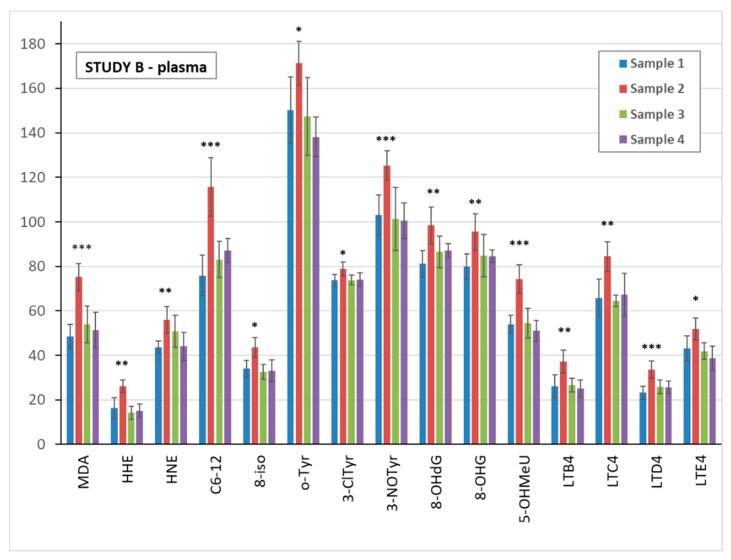
Comparison of the mean levels of oxidative stress and inflammatory markers in study B blood plasma pre-exposure (sample 1) and after ultra violet (UV) exposure (samples 2–4). * (*p* < 0.05) ** (*p* < 0.01) *** (*p* < 0.001). MDA = malondialdehyde, HHE = 4-hydroxy-*trans*-hexenal, HNE = 4-hydroxy-*trans*-nonenal, C6-12 = aldehydes C6-C12, (all ng/mL); 8-iso = 8-isoProstaglandin F2α, o-Tyr = o-tyrosine, 3-ClTyr = 3-chlorotyrosine, 3-NOTyr = 3-nitrotyrosine, 8-OHdG = 8-hydroxy-2-deoxyguanosine, 8-OHG = 8-hydroxyguanosine, 5-OHMeU = 5-hydroxymethyl uracil, and leukotrienes (LT) LTB4, LTC4, LTD4, LTE4 (all pg/mL).

**Figure 4:**
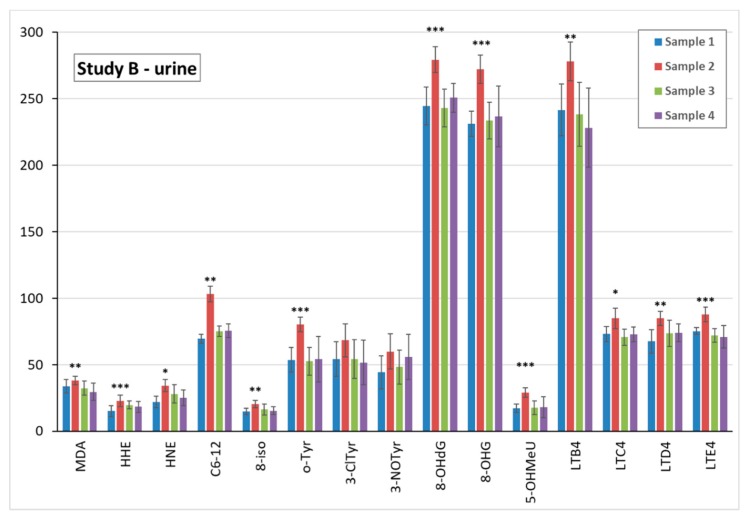
Comparison of the mean levels of oxidative stress and inflammatory markers in study B urine pre-exposure (sample 1) and after UV exposure (samples 2–4). * (*p* < 0.05) ** (*p* < 0.01) *** (*p* < 0.001). MDA = malondialdehyde, HHE = 4-hydroxy-*trans*-hexenal, HNE = 4-hydroxy-*trans*-nonenal, C6-12 = aldehydes C6-C12, (all ng/mL); 8-iso = 8-isoProstaglandin F2α, 8-OHdG = 8-hydroxy-2-deoxyguanosine, 8-OHG = 8-hydroxyguanosine, 5-OHMeU = 5-hydroxymethyl uracil, o-Tyr = o-tyrosine, 3-ClTyr = 3-chlorotyrosine, 3-NOTyr = 3-nitrotyrosine, leukotrienes (LT) LTB4, LTC4, LTD4, LTE4 (all pg/mL).

UV exposure in study B significantly elevated all of the biomarkers analysed in sample 2 of plasma, urine, and EBC, as compared with pre-exposure sample 1. Data are shown in Figure 3 for plasma, Figure 4 for urine, and Supplementary Figure S1 for EBC samples.

**Figure 5:**
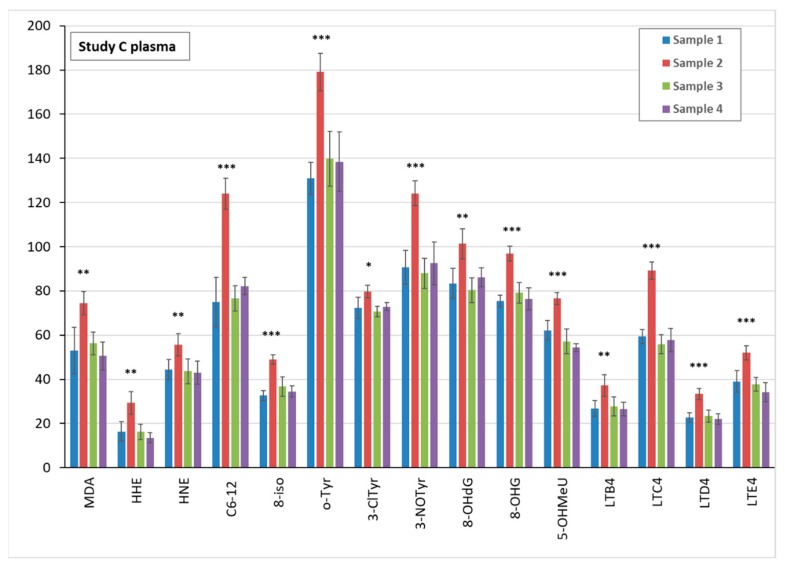
Comparison of the mean levels of oxidative stress and inflammatory markers in study C blood plasma pre-exposure (sample 1) and after both sunscreen and UV exposure (samples 2–4). * (*p* < 0.05) ** (*p* < 0.01) *** (*p* < 0.001). MDA = malondialdehyde, HHE = 4-hydroxy-*trans*-hexenal, HNE = 4-hydroxy-*trans*-nonenal, C6-12 = aldehydes C6-C12, (all ng/mL); 8-iso = 8-isoProstaglandin F2α, o-Tyr = o-tyrosine, 3-ClTyr = 3-chlorotyrosine, 3-NOTyr = 3-nitrotyrosine, 8-OHdG = 8-hydroxy-2-deoxyguanosine, 8-OHG = 8-hydroxyguanosine, 5-OHMeU = 5-hydroxymethyl uracil, and leukotrienes (LT) LTB4, LTC4, LTD4, LTE4 (all pg/mL).

Moreover, in study C, when sunscreen was used prior to UV irradiation, a significant elevation in all biomarkers in plasma sample 2 was found, as presented in Figure 5.

Individual levels of the biomarkers in study B and study C and their comparison is shown in Supplementary Table S1. There were few differences in the biological sample’s levels, however no marker decreased significantly in samples 2 when the sunscreen was used.

**Figure 6:**
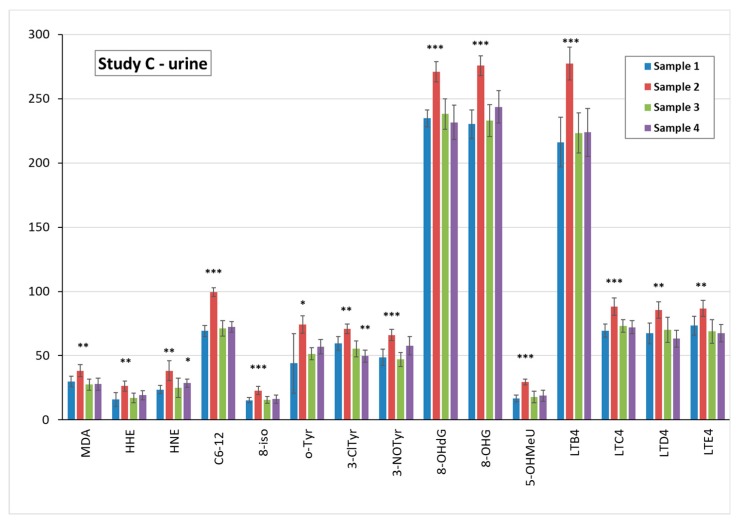
Comparison of the mean levels of oxidative stress and inflammatory markers in study C exhaled breath condensate pre-exposure (sample 1) and after both sunscreen and UV exposure (samples 2–4). * (*p* < 0.05) ** (*p* < 0.01) *** (*p* < 0.001). MDA = malondialdehyde, HHE = 4-hydroxy-*trans*-hexenal, HNE = 4-hydroxy-*trans*-nonenal, C6-12 = aldehydes C6-C12, (all ng/mL); 8-iso = 8-isoProstaglandin F2α, 8-OHdG = 8-hydroxy-2-deoxyguanosine, 8-OHG = 8-hydroxyguanosine, 5-OHMeU = 5-hydroxymethyl uracil, o-Tyr = o-tyrosine, 3-ClTyr = 3-chlorotyrosine, 3-NOTyr = 3-nitrotyrosine, leukotrienes (LT) LTB4, LTC4, LTD4, LTE4 (all pg/mL).

Accordingly, in urine, all 15 biomarkers in sample 2 were elevated, as can be seen in Figure 6.

The results of the EBC samples in study C show an increase that is less uniform. Among the 8 elevated markers of the oxidation of lipids, MDA, HHE, and HNE peaks were postponed in sample 3, as can be seen in Supplementary Figure S2. A significant elevation in sample 2 was observed in all markers of the oxidation of nucleic acids, and in LTD4 and LTE4. With respect to the remaining markers, the difference was not significant, however a positive trend could still be observed. It can be seen that the variability (i.e., confidence intervals) of the biomarkers was broader in the EBC samples as compared with both the plasma and urine samples.

The significance level of the biomarkers in the plasma in study C (9 × *p* < 0.001, 5 × *p* < 0.01 and 1 × *p* < 0.05) was no lower than that in study B (5 × *p* < 0.001, 7 × *p* < 0.01 and 3 × *p* < 0.05), including markers of the oxidation of nucleic acids. This level was similar in urine samples.

### 3.6. TiO_2_ Concentration and Particle Analysis in Plasma, Spot Urine, and EBC

#### 3.6.1. TiO_2_ Concentration in Biological Samples

TiO_2_ was detected solely in tests using the sunscreen (studies A and C) and only in samples 2, 3, and 4. The results are shown in Table 2 and Table 3. Measurable TiO_2_ was found only in the plasma and urine. In all EBC samples, TiO_2_ was below the LOD.

Comparison of the mean titania concentrations in studies A and C, i.e., sunscreen without and with UV exposure respectively, in plasma and urine samples showed that the difference was not significant. In the plasma samples in studies A and C, the average titania concentration was 8.9 ± 2.8 ng/mL and 9.6 ± 3.4 ng/mL respectively (*p* = 0.838), and that in urine samples was 6.7 ± 1.6 ng/mL and 6.6 ± 1.7 ng/mL, respectively (*p* = 0.961).

As can be seen in Table 2 and Table 3, TiO_2_ could be measured in women in both test variants in samples 2, 3, and 4, but in men only in samples 3 and 4. The highest measured levels were found in sample 3 from women.

When the results from men and women were calculated separately, the average concentration of titania in all plasma samples in study A was significantly higher in women (10.90 ± 1.55 mg/mL) than that in men (3.97 ± 2.89 mg/mL) (*p* = 0.0216). Moreover, in study C, the average plasma titania concentration was significantly higher in women (12.13 ± 1.44 mg/mL) than that in men (3.80 ± 2.81 mg/mL) (*p* = 0.0103).

#### 3.6.2. Transmission Electron Microscopy (TEM) Analysis of TiO_2_ Particles in Plasma, Urine, and EBC

In agreement with the titania results, no particles were found by TEM in the biological samples collected prior to or without sunscreen application, i.e., in sample 1 or in all samples in study B (only UV irradiation).

**Figure 7:**
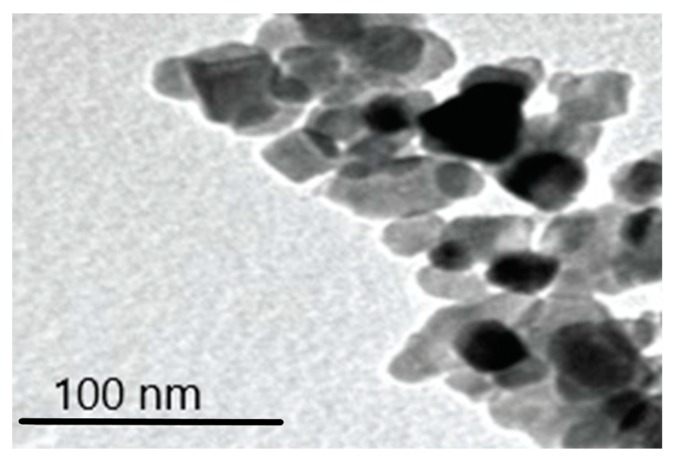
Typical transmission electron microscopy (TEM) micrograph of TiO_2_ particles in plasma samples. Magnification bar = 100 nm.

TEM micrographs prove the presence of TiO_2_ particles in the plasma (Figure 7) during sunscreen use and one week after sunscreen removal in studies A and C.

**Figure 8:**
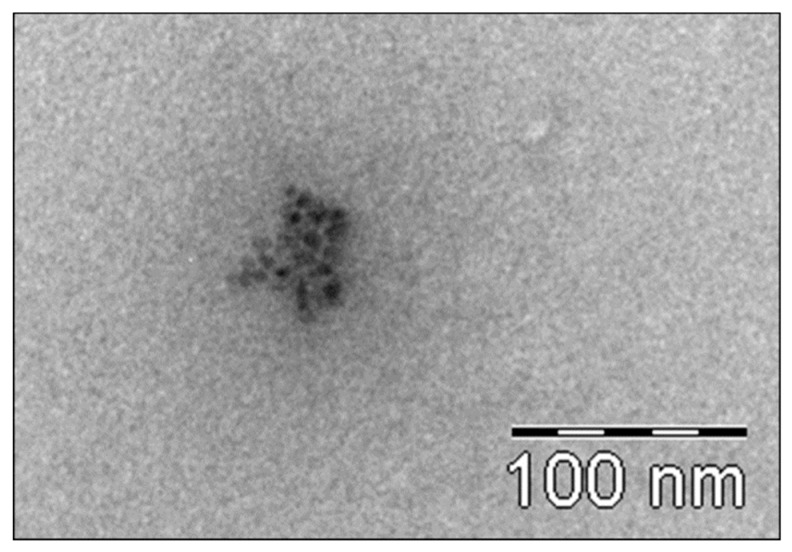
Transmission electron microscopy (TEM) micrograph of TiO_2_ nanoparticles in the urine (with agglomeration of particles). Magnification bar = 100 nm.

**Figure 9:**
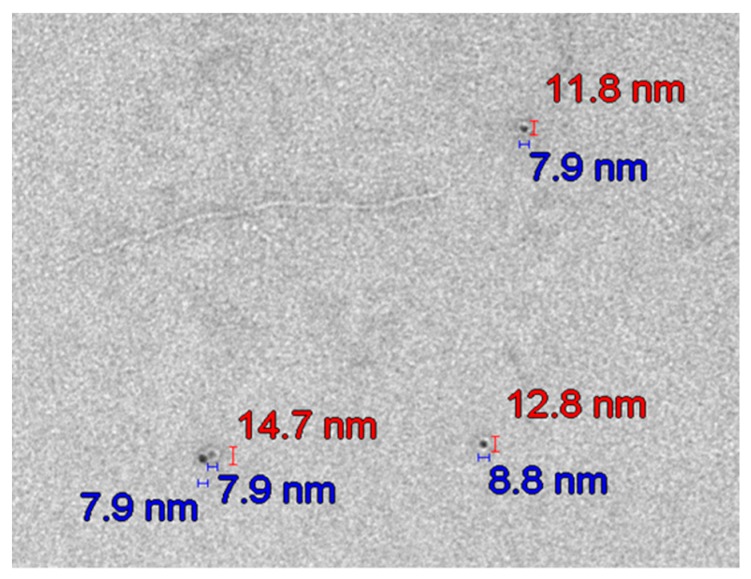
Typical transmission electron microscopy (TEM) micrograph of the size of TiO_2_ nanoparticles in urine in study A. The particles are not ideally spherical and thus are characterised by two dimensions (red and blue abscissas in the micrograph).

Figure 8 and Figure 9 show the typical transmission electron Microscopy (TEM) micrographs in urine, with or without agglomeration of TiO_2_ nanoparticles, respectively. No difference was noted with respect to the particle size between studies A and C.

The average particle size in plasma samples 2, 3, and 4 in studies A and C increased with time, and the largest average particle size was found in sample 4. The same trend was seen for the urine samples in the same studies, as presented in Table 4 and Table 5.

No significant difference between the mean particle size in plasma samples was seen when sunscreen-applied skin was UV-irradiated (study A: 7.4 ± 0.6 nm versus study C: 7.1 ± 0.7 nm; *p* = 0.879). Moreover, no difference was found in spot urine samples between studies A and C (5.8 ± 0.5 nm versus 5.6 ± 0.5 nm, respectively; *p* = 0.876). The mean values are shown in Table 4 and Table 5, respectively.

**Figure 10:**
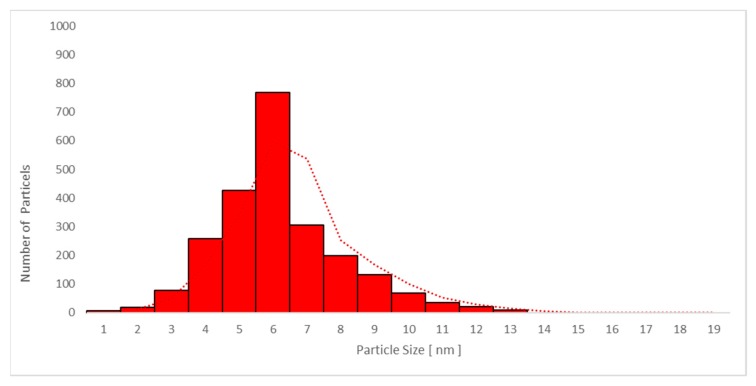
Particle size distribution detected using transmission electron microscopy (TEM) in plasma sample 2 in study C (sunscreen + UV). The average particle size was 6.1 nm (detected only in women due to faster absorption as compared with men).

**Figure 11:**
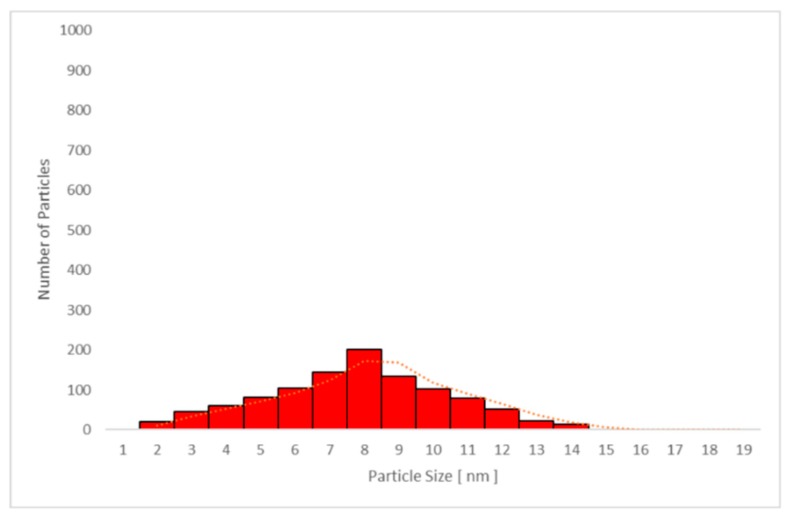
Particle size distribution detected using transmission electron microscopy (TEM) in plasma sample 4 in study C (sunscreen + UV) (one week after sunscreen removal). The average particle size was 7.8 nm in all subjects.

**Figure 12:**
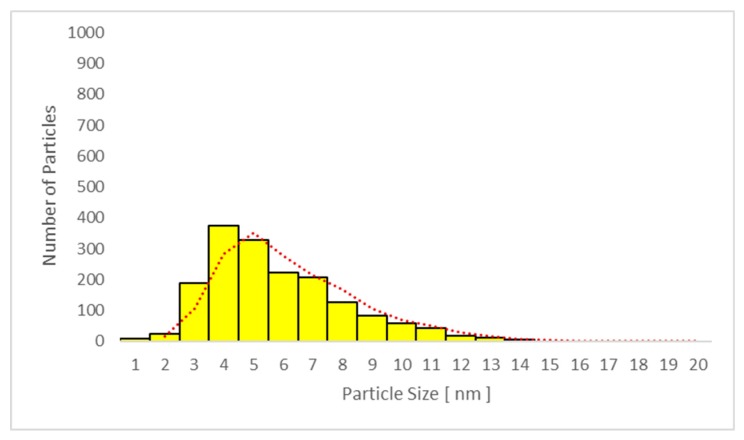
Particle size distribution determined by transmission electron microscopy (TEM) in urine sample 3 in study C (sunscreen + UV). The average particle size was 5.4 nm in all subjects.

**Figure 13:**
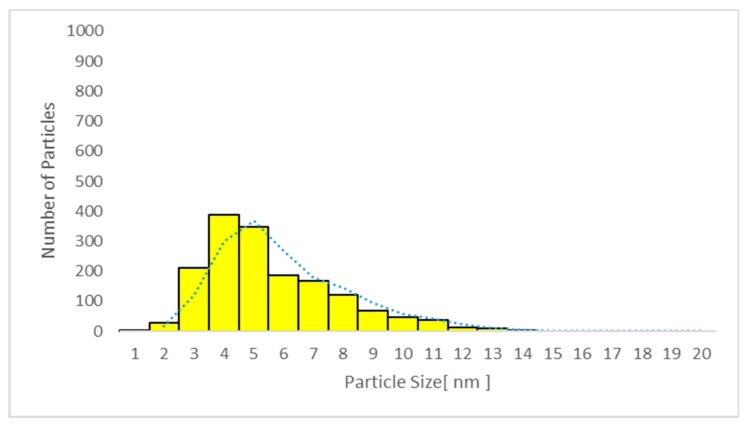
Particle size distribution determined by transmission electron microscopy (TEM) in urine sample 4 in study C (sunscreen + UV). The average particle size was 6.2 nm in all subjects.

Only particles sized 1–14 nm were found in the plasma (Figure 10 and Figure 11) and urine spot (Figure 12 and Figure 13) samples in studies A and C in which sunscreen was used. The gliding trend fit was interleaved and is presented in these figures.

### 3.7. FeNO

No FeNO level reached 50 ppb, and the mean group levels in samples 2, 3, and 4 did not differ significantly from the average sample 1 in any study variant (data not shown).

### 3.8. Spirometry

Lung function parameters were within the reference range in most subjects, solely a borderline impairment of FEV1% reaching 72%–75% of the reference range was found in the subject being treated for asthma at all time intervals and in all three test variants. However, there were no significant changes in spirometry parameters among the three study variants (data not shown).

## 4. Discussion

There were five key findings of the present study. Firstly, a potentially damaging effect of sunscreen was not found. We did not see elevation in the markers of oxidative stress and inflammation in any sample—plasma, urine, or EBC—after using sunscreen for three consecutive days or one week after their thorough removal from the skin. This indicates that even a longer usage of sunscreen than is common, i.e., without washing off the sunscreen each evening, was not detrimental.

There was an elevation in a few biomarkers in the EBC in study A, as compared with pre-test levels, however, due to the higher variability of the results and technical challenges of EBC collection and analysis, and since EBC is not yet considered a routine clinical method, we do not want to overestimate their significance.

Secondly, a beneficial effect of sunscreen application was not seen, since the levels of biomarkers of oxidative stress and inflammation remained unchanged following UV radiation. The sunscreen was applied prior to UV exposure as recommended, and the prescribed dose was used (less than 140 g on average).

On one hand, the sunscreen protected the skin from mild redness as seen in study B. However, on the other hand, the sunscreen did not block/prevent the increase in markers of systemic oxidative stress and inflammation in the test variant C, i.e., sunscreen + UV (conditions for which the use of sunscreen is recommended) as compared with UV only. Both plasma and urine samples reflected a rapid effect in both studies.

With respect to the EBC, the number of positive samples in study C was lower, and the peak elevation of three markers of the oxidation of lipids was postponed to sample 3, i.e., it was seen later, and although there was an increasing trend, significance was not seen in 8-iso, o-Tyr, 3-ClTyr, 3-NOTyr, and LTC4. EBC usually shows a higher variability and may encounter more interfering factors in the respiratory system [38]. Therefore, the limited number of subjects may have played a role. Although spirometry and FeNO were within the reference values during all test variants and in all samples, subclinical involvement cannot be excluded [38].

The length of sunscreen use in the present study was longer than common use by consumers. The difference was that the sunscreen was not washed off in the evenings on days 1, 2, and 3. Nevertheless, the dosage corresponded to the amount recommended by the manufacturer, which was approximately 30 g twice per day per adult and re-application following accidental removal due to swimming or towelling. The total recommended sunscreen amount for three days (180 g) was not exceeded.

Thirdly, there was a measurable titania concentration and the detection of TiO_2_ particles using TEM in plasma and urine samples in studies A and C, which supports the theory of the skin permeability potential of nanoTiO_2_.

The sunscreen use duration was three days, and the sunscreen was not washed off at the end of each day, which differs from regular use of the sunscreen. However, the sunscreen dose did not exceed the recommended dose for the population, including children. Similarly, the UV irradiation dose was within the EU limits for commercial tanning beds.

Only nanoparticles up to a diameter of 14 nm were detected in both biological fluids. The average particle size in plasma showed an increase over time from 6.1 nm to 7.8 nm, i.e., larger particles persisted longer. Both measurable titania and nanoparticles were present up to sample 4, i.e., one week after washing the sunscreen off, which excludes contamination from the sunscreen that was thoroughly washed off one week earlier. Of course, urine contamination could not be completely ruled out, however plasma contamination would not occur during standard blood withdrawal.

Fourthly, we could exclude respiratory delivery of nanoTiO_2_ to the body based on the negative results of both TiO_2_ and TEM particle analysis of EBC samples.

Negative TiO_2_ in the biological samples without sunscreen application also excludes food as a potential source of nanoTiO_2._ Potential contamination between skin-hands-mouth cannot be completely excluded, however the absorption from the gastrointestinal tract is very limited [50], therefore we do not suppose this could bring measurable plasma and urine levels. In addition, we have no explanation as to why it would be more pronounced in females. We would also expect that the nanoparticles, the origin of which would be the skin-hands-mouth contamination, would keep the original size, i.e., of approximately 43 nm, as oral absorption enables absorption of larger particles, with an average that was much higher than in our plasma and urine samples. In human volunteers’ oral exposure of 100-mg dose of TiO_2_ particles with diameter 50–260 nm appeared in the blood [51].

Fifthly, there was an early appearance of TiO_2_ in both blood and urine under normal conditions of sunscreen use, since titania and TiO_2_ particles were found 6 h after the first sunscreen use in sample 2 from women. This shows that the continuous use of sunscreen did not affect these results.

This was seen later in men, i.e., in sample 3 collected 42 h later. Since there was no sampling on day 2 and both plasma and urine were actually spot samples and not 24-h collection, it is possible that titania appeared in the samples from men earlier than day 3, when the highest levels were measured in both women and men.

Our study supports the findings by Gulson et al., using sunscreen containing nanoparticles of ^68^ZnO, in which the tracer was found in blood and urine samples at the end of the second day when sunscreen was used twice per day [52]. In their 5-day studies with sunscreen applied to a small area of the skin, such as the mid to upper back, ^68^Zn positivity continued to increase for up to 9 days after the end of application [52,53].

In the present study, the concentration of titania in the plasma from women was significantly higher in both studies A and C, although the dose per body surface was not significantly higher than that in men. Titania may have been absorbed by inter- or intra-cellular diffusion, through hair follicles, sweat glands, and skin folds, or a combination. The thinner skin in women relative to men has been suggested as one reason [53]. Other factors, such as hormone metabolism, hair growth, sweat rate, sebum production, and fat accumulation, should probably be considered [54]. Here also, the formulation of the sunscreen contained the chelating agent, EDTA, which may potentially bind Ti and promote its absorption [53].

The penetration of nanoparticles beyond the stratum corneum suggests that oxidative stress may lead to more serious adverse cellular effects, and potentially cancer [43]. However, similar to the nanoZnO study, the quantity absorbed was very low.

We did not see any additional effect of UV irradiation (study C) as compared with sunscreen only (study A) on the mean level of TiO_2_ and nanoparticles in plasma and urine.

To the best of our knowledge, this is the first study to provide evidence that TiO_2_ nanoparticles in sunscreen are absorbed through healthy human skin, both exposed and unexposed to UV radiation, and that these particles are detectable in blood and urine.

More data are available concerning the inhalation of nanoTiO_2_ particles, especially in workers with long-term exposure to TiO_2_ dust, where particles documented by Raman microspectroscopy of rutile and/or anatase originating from preceding shifts were found in their EBC [55]. TiO_2_ in EBC reached an average of 24 ng/mL, in contrast to the controls, where it was unmeasurable, similar to the present sunscreen study [55]. Recently, other methods of nanoparticle detection have been successfully used [56,57]. Due to the potential of sunscreen in the spray form to be absorbed by inhalation, such products have been classified as potentially deleterious and are no longer allowed [58].

Elevated markers of oxidative stress, including oxidation products of lipids, proteins, and nucleic acids, are associated with aging, metabolic diseases [40,59], exposure to carcinogens [60,61,62], and especially cancer [13,14], however, the observed effects on oxidative stress and inflammation are unknown. A study in rats exposed to nanoTiO_2_ by inhalation for four weeks showed a strong inflammatory response in bronchoalveolar lavage peaking on day 3 post-exposure, but the overexpression of genes involved in inflammation was maintained for six months after the end of exposure [63]. Long-term response was characterised by persistent upregulation of a number of genes, including those involved in oxidative stress, for up to 180 days post-exposure [63].

The main limitation of the present pilot study is the relatively low number of exposed subjects, which is a consequence of the high financial demands of such studies [23]. Only one commercial type of nanoTiO_2_ sunscreen was used. In addition, this pilot study did not analyse skin samples. Such a study could bring important data, especially in repeated samples. The advantage, on the other hand, is the fact that the participating subjects acted as their own controls and were identical during all three study variants, which eliminates intra-individual variability. Although direct measurement of UV exposure was not possible, the use of UV exposure in the form of a tanning bed with a defined ceiling limit for the intensity and an exact duration enabled achievement of a comparable UV dose for all subjects, not influenced by the weather or location conditions.

Spirometry and FeNO measurements did not show any significant differences and helped to exclude significant respiratory effects and impairments.

## 5. Conclusions

Sunscreen alone did not cause an elevation in the vast majority of the biomarkers of oxidative stress and inflammation, however, tanning bed use increased all markers in plasma, urine, and EBC. A preventive effect of sunscreen was not found. Its use prior to UV irradiation suppressed skin redness, however, sunscreen did not prevent the effects of significantly elevated oxidative stress and inflammatory markers, including those of nucleic acid oxidation. Plasma or urine analysis appears preferred to EBC due to the lower potential interference of inhalation.

Titania and nanoTiO_2_ particles were found in plasma and spot urine samples from women after 6 h of sunscreen use, but in males after 48 h. We show that nanoTiO_2_ particles can pass through the protective layers of the skin even without UV irradiation, and can be detected in blood and urine up to one week after removal of the sunscreen by bathing and showering. Measurable titania levels and TiO_2_ nanoparticles prove absorption and exclude potential contamination. No additional effect of the UV irradiation on the absorption of sunscreen was seen.

Positive plasma and urine TiO_2_ measurements and TEM particle detection during nanoTiO_2_ sunscreen use confirm that a minor amount is absorbed and slowly eliminated. The amounts were minor, and TiO_2_ detected in the blood and urine from sunscreen may not necessarily be present in the form of TiO_2_ nanoparticles.

Importantly, the absence of both titania and TiO_2_ particles in the EBC by ICP-MS and TEM respectively, excluded the inhalation of TiO_2_.

The present pilot study doubts the utility and positive effect of nanoTiO_2_ sunscreen to prevent oxidative stress and inflammation caused by UV irradiation, suggesting that it may not prevent skin cancers.

In addition, it appears that nanoparticles can be absorbed through the skin and pass to the urine, starting 6 h after first exposure, and can remain for up to one week after the sunscreen has been washed off the skin.

Since there is an increasing concern regarding the exposure of pregnant mothers, women, men of fertile age, and notably children to nanoTiO_2_ [64], further human studies are needed to explain the impact of the elevated markers used in the present study. Also, our hypothesis that only particles with a diameter size lower than approximately 15 nm may be absorbed through the skin should be verified, as replacing them in the sunscreen with larger particles could potentially prevent absorption.

## Figures and Tables

**Table 1:** Schedule of the three study variants, exposure times, and sampling of the blood plasma, spot urine, and exhaled breath condensate (EBC).

	Day 1			Day 3	Day 4	Day 11
Sample	Sample 1 Pre-test (time)	Tests (time)	Sample 2 (time)	Tests (time)	Sample 3 (time)	Sample 4 Post-test (time)
**Study A** Sunscreen only (December 2014)	8:00	9:00 Sunscreen continuously	14:00	Sunscreen continuously	8:00 (8:30 Sunscreen washed off)	8:00
**Study B** UV only (June 2015)	8:00	11:30 UV 1 (8 min)	14:00	11:30 UV 2 (8 min)	8:00	8:00
**Study C** Sunscreen + UV (March 2015)	8:00	9:00 Sunscreen continuously	14:00	Sunscreen continuously	8:00 (8:30 Sunscreen washed off)	8:00
		11:30 UV 1 (8 min)		11:30 UV 2 (8 min)		

**Table 2:** TiO_2_ concentration measured in the plasma, spot urine, and exhaled breath condensate (EBC) in study A (sunscreen only). Limit of detection (LOD) 1.2 ng/mL.

Sample No/Group	TiO_2_ Plasma (ng/mL)	TiO_2_ Urine (ng/mL)	TiO_2_ EBC (ng/mL)
1 All subjects	<LOD	<LOD	<LOD
2 Men	<LOD	<LOD	<LOD
2 Women	9	5.8	<LOD
3 Men	6.8	6.9	<LOD
3 Women	12.8	9.3	<LOD
4 Men	5.1	4.5	<LOD
4 Women	10.9	7.2	<LOD
All samples **Mean ± SD**	**8.9 ± 2.8**	**6.7 ± 1.6**	**<LOD**

**Table 3:** TiO_2_ concentration measured in plasma, spot urine, and exhaled breath condensate (EBC) in study C (sunscreen + UV irradiation). Limit of detection (LOD) 1.2 ng/mL.

Sample No/Group	TiO_2_ Plasma (ng/mL)	TiO_2_ Urine (ng/mL)	TiO_2_ EBC(ng/mL)
All subjects	<LOD	<LOD	<LOD
2 Men	<LOD	<LOD	<LOD
2 Women	11.6	5.5	<LOD
3 Men	6.7	7.2	<LOD
3 Women	14.1	9.3	<LOD
4 Men	4.7	4.4	<LOD
4 Women	10.7	6.8	<LOD
All samples **Mean ± SD**	**9.6 ± 3.4**	**6.6 ± 1.7**	**<LOD**

**Table 4:** Particle size distribution (PSD) of TiO_2_ measured in plasma, spot urine, and exhaled breath condensate (EBC) in study A (sunscreen). Resolution 1 nm.

Sample No/Group	Average Particle Size TiO_2_ Plasma (nm)	Average Particle Size TiO_2_ Urine (nm)	Average Particle Size TiO_2_ EBC (nm)
1 All subjects	<LOD	<LOD	<LOD
2 Men	<LOD	<LOD	<LOD
2 Women	6.5	5.4	<LOD
3 Men	7.2	5.2	<LOD
3 Women	7.5	6.1	<LOD
4 Men	7.6	5.6	<LOD
4 Women	8.4	6.7	<LOD
All samples **Mean ± SD**	**7.4 ± 0.6**	**5.8 ± 0.5**	**<LOD**

**Table 5:** Particle size distribution (PSD) of TiO_2_ measured in plasma, spot urine, and exhaled breath condensate (EBC) in study C (sunscreen + UV irradiation). Resolution 1 nm.

Sample No/Group	TiO_2_ Plasma (nm)	TiO_2_ Urine (nm)	TiO_2_ EBC (nm)
1 All subjects	<LOD	<LOD	<LOD
2 Men	<LOD	<LOD	<LOD
2 Women	6.1	5.2	<LOD
3 Men	6.8	5.0	<LOD
3 Women	7.2	5.8	<LOD
4 Men	7.4	5.4	<LOD
4 Women	8.2	6.4	<LOD
All samples **Mean ± SD**	**7.1 ± 0.7**	**5.6 ± 0.5**	**<LOD**

## Supplementary Materials

The following are available online at https://www.mdpi.com/2079-4991/9/6/888/s1. Figure S1. Comparison of the mean levels of oxidative stress and inflammatory markers in study B exhaled breath condensate (EBC) pre-exposure (sample 1) and after UV exposure (samples 2–4). * (*p* < 0.05) ** (*p* < 0.01) *** (*p* < 0.001). MDA = malondialdehyde, HHE = 4-hydroxy-*trans*-hexenal, HNE = 4-hydroxy-*trans*-nonenal, C6-12 = aldehydes C6-C12, (all ng/mL); 8-iso = 8-isoProstaglandin F2α, o-Tyr = o-tyrosine, 3-ClTyr = 3-chlorotyrosine, 3-NOTyr = 3-nitrotyrosine, 8-OHdG = 8-hydroxy-2-deoxyguanosine, 8-OHG = 8-hydroxyguanosine, 5-OHMeU = 5-hydroxymethyl uracil, and leukotrienes (LT) LTB4, LTC4, LTD4, LTE4 (all pg/mL). Figure S2. Comparison of the mean levels of oxidative stress and inflammatory markers in study C exhaled breath condensate (EBC) pre-exposure (sample 1) and after both sunscreen and UV exposure (samples 2–4). * (*p* < 0.05) ** (*p* < 0.01) *** (*p* < 0.001). MDA = malondialdehyde, HHE = 4-hydroxy-*trans*-hexenal, HNE = 4-hydroxy-*trans*-nonenal, C6-12 = aldehydes C6-C12, (all ng/mL); 8-iso = 8-isoProstaglandin F2α, o-Tyr = o-tyrosine, 3-ClTyr = 3-chlorotyrosine, 3-NOTyr = 3-nitrotyrosine, 8-OHdG = 8-hydroxy-2-deoxyguanosine, 8-OHG = 8-hydroxyguanosine, 5-OHMeU = 5-hydroxymethyl uracil, and leukotrienes (LT) LTB4, LTC4, LTD4, LTE4 (all pg/mL). Table S1. Individual and means levels of markers of oxidative stress and inflammation in plasma samples; and comparison of the study B (UV only) and C (sunscreen + UV). MDA = malondialdehyde, HHE = 4-hydroxy-*trans*-hexenal, HNE = 4-hydroxy-*trans*-nonenal, C6-12 = aldehydes C6-C12, (all ng/mL); 8-iso = 8-isoProstaglandin F2α, o-Tyr = o-tyrosine, 3-ClTyr = 3-chlorotyrosine, 3-NOTyr = 3-nitrotyrosine, 8-OHdG = 8-hydroxy-2-deoxyguanosine, 8-OHG = 8-hydroxyguanosine, 5-OHMeU = 5-hydroxymethyl uracil, and leukotrienes (LT) LTB4, LTC4, LTD4, LTE4 (all pg/mL). * (*p* < 0.05) ** (*p* < 0.01).
