# Supplementary material for: NanoTiO2 Sunscreen Does Not Prevent Systemic Oxidative Stress Caused by UV Radiation and a Minor Amount of NanoTiO2 is Absorbed in Humans
Source: Nanomaterials (Basel). 2019 Jun 17;9(6):888. doi: 10.3390/nano9060888 (PMC6631994; doi:10.3390/nano9060888)
Supplement: Supplementary file 1 [file nanomaterials-09-00888-s001.pdf]

Supplementary Materials

NanoTiO2 Sunscreen Does Not Prevent Systemic Oxidative Stress Caused by UV Radiation and a Minor Amount of NanoTiO2 is Absorbed in Humans

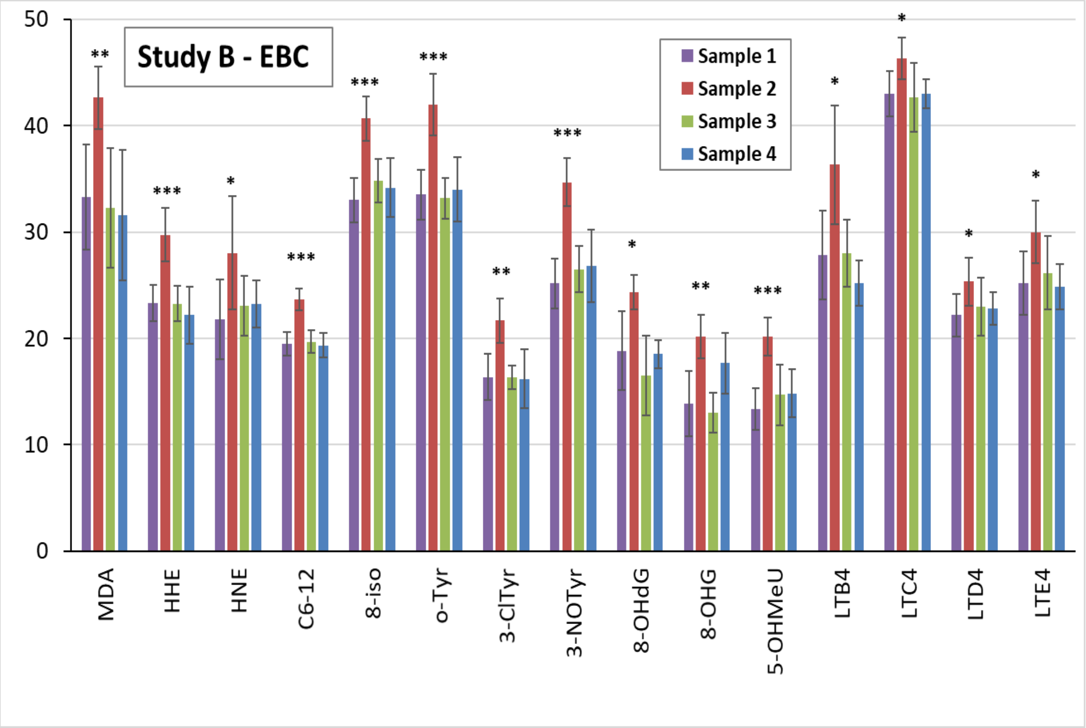

Figure S1. Comparison of the mean levels of oxidative stress and inflammatory markers in study B exhaled breath condensate (EBC) pre-exposure (sample 1) and after UV exposure (samples 2–4). *($p<0.05$) **($p<0.01$) ***($p<0.001$).

MDA=malondialdehyde, HHE=4-hydroxy-trans-hexenal, HNE=4-hydroxy-trans-nonenal, C6-12=aldehydes C6-C12, (all ng/mL); 8-iso=8-isoProstaglandin F2α, o-Tyr=o-tyrosine, 3-ClTyr=3-chlorotyrosine, 3-NOTyr=3-nitrotyrosine, 8-OHdG=8-hydroxy-2-deoxyguanosine, 8-OHG=8-hydroxyguanosine, 5-OHMeU=5-hydroxymethyl uracil, and leukotrienes (LT) LTB4, LTC4, LTD4, LTE4 (all pg/mL).

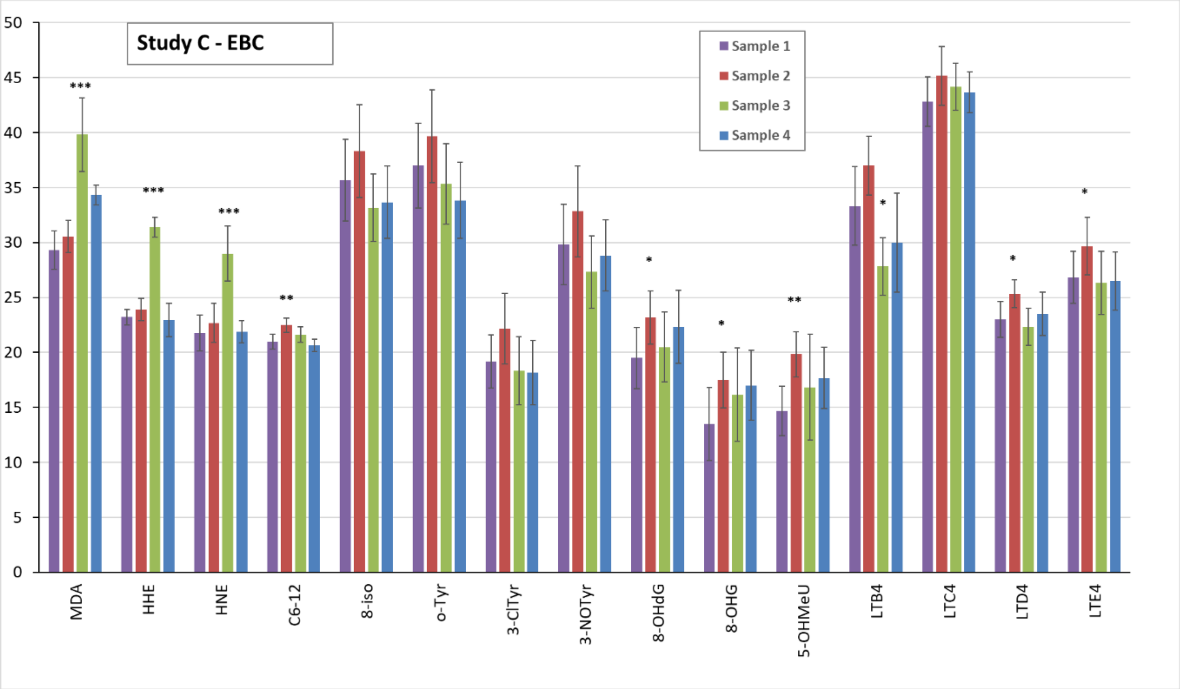

Figure S2. Comparison of the mean levels of oxidative stress and inflammatory markers in study C exhaled breath condensate (EBC) pre-exposure (sample 1) and after both sunscreen and UV exposure (samples 2–4). *(p<0.05) **(p<0.01) ***(p<0.001).

MDA=malondialdehyde, HHE=4-hydroxy-trans-hexenal, HNE=4-hydroxy-trans-nonenal, C6-12=aldehydes C6-C12, (all ng/mL); 8-iso=8-isoProstaglandin F2α, o-Tyr=o-tyrosine, 3-ClTyr=3-chlorotyrosine, 3-NOTyr=3-nitrotyrosine, 8-OHdG=8-hydroxy-2-deoxyguanosine, 8-OHG=8-hydroxyguanosine, 5-OHMeU=5-hydroxymethyl uracil, and leukotrienes (LT) LTB4, LTC4, LTD4, LTE4 (all pg/mL).

Table S1. Individual and means levels of markers of oxidative stress and inflammation in plasma samples; and comparison of the study B (UV only) and C (sunscreen + UV). MDA=malondialdehyde, HHE=4-hydroxy-trans-hexenal, HNE=4-hydroxy-trans-nonenal, C6-12=aldehydes C6-C12, (all ng/mL); 8-iso=8-isoProstaglandin F2α, o-Tyr=o-tyrosine, 3-ClTyr=3-chlorotyrosine, 3-NOTyr=3-nitrotyrosine, 8-OHdG=8-hydroxy-2-deoxyguanosine, 8-OHG=8-hydroxyguanosine, 5-OHMeU=5-hydroxymethyl uracil, and leukotrienes (LT) LTB4, LTC4, LTD4, LTE4 (all pg/mL). *(p<0.05) **(p<0.01)

**Sample No.**	**Sample 1**		**Sample 2**		**Sample 3**		**Sample 4**	
**Marker/ Study**	**MDA/B**	**MDA/C**	**MDA/B**	**MDA/C**	**MDA/B**	**MDA/C**	**MDA/B**	**MDA/C**
**Female**	41	44	78	69	61	52	53	55
**Male**	53	42	68	80	46	63	43	53
**Male**	52	47	69	81	56	57	59	48
**Male**	48	57	77	79	48	51	57	48
**Female**	44	66	80	74	65	61	55	41
**Female**	53	62	75	68	48	54	41	58
**Mean±SD**	**48.5±5.3**	**53±11**	**74.5±5.2**	**75.2±6**	**54±8.3**	**56.3±5.1**	**51.3±7.9**	**50.5±6.4**
p	0.392		0.847		0.586		0.851	

**Sample No.**	**Sample 1**		**Sample 2**		**Sample 3**		**Sample 4**	
**Marker/ Study**	**HHE/B**	**HHE/C**	**HHE/B**	**HHE/C**	**HHE/B**	**HHE/C**	**HHE/B**	**HHE/C**
**Female**	14	13	23	25	17	13	11	12

**Male**	24	12	26	24	18	12	17	13
**Male**	11	15	25	34	11	17	16	14
**Male**	16	21	25	36	12	16	19	17
**Female**	16	22	26	30	14	18	13	11
**Female**	17	15	31	27	13	21	15	14
**Mean±SD**	**16.3±4.5**	**16.3±4.4**	**26±2.8**	**29.3±5.1**	**14.2±2.9**	**16.2±3.5**	**15.2±3**	**13.5±2.2**
p	1.000		0.211		0.326		0.316	

**Sample No.**	**Sample 1**		**Sample 2**		**Sample 3**		**Sample 4**	
**Marker/ Study**	**HNE/B**	**HNE/C**	**HNE/B**	**HNE/ C**	**HNE/B**	**HNE/C**	**HNE/B**	**HNE/C**
**Female**	40	41	46	55	57	46	43	39
**Male**	44	40	56	62	54	46	38	44
**Male**	48	43	58	52	41	41	39	51
**Male**	42	46	60	50	45	47	44	42
**Female**	45	45	54	54	50	48	45	45
**Female**	43	52	62	61	58	34	55	37
**Mean±SD**	**43.7±2.9**	**44.5±4.5**	**56±5.9**	**55.7±5.1**	**50.8±7.1**	**43.7±5.6**	**44±6.4**	**43±5.2**
p	0.723		0.922		0.093		0.781	

**Sample No.**	**Sample 1**		**Sample 2**		**Sample 3**		**Sample 4**	
**Marker/ Study**	**C6-12/B**	**C6-12/C**	**C6-12/B**	**C6-12/C**	**C6-12/B**	**C6-12/C**	**C6-12/B**	**C6-12/C**
**Female**	74	70	92	115	81	73	90	83
**Male**	86	70	123	120	83	83	95	81
**Male**	66	68	116	127	72	80	85	87
**Male**	70	80	128	134	96	75	83	85
**Female**	71	67	114	127	85	80	89	81
**Female**	87	94	122	122	82	69	81	76
**Mean±SD**	**75.9±9.1**	**75±11**	**116±13**	**124±7.1**	**83.1±8.1**	**76.7±5.7**	**87.1±5.4**	**82.2±4**
p	0.880		0.223		0.167		0.117	

**Sample No.**	**Sample 1**		**Sample 2**		**Sample 3**		**Sample 4**	
**Marker/ Study**	**8-iso/B**	**8-iso/C**	**8-iso/B**	**8-iso/C**	**8-iso/B**	**8-iso/C**	**8-iso/B**	**8-iso/C**
**Female**	35	32	41	46	32	38	32	37
**Male**	38	33	49	49	35	40	38	33
**Male**	30	36	37	52	31	31	37	31
**Male**	38	31	45	50	35	33	26	34
**Female**	31	30	44	48	27	42	36	37
**Female**	32	34	46	48	35	36	30	35
**Mean±SD**	**34±3.7**	**32.7±2.3**	**43.7±4.4**	**48.8±2.1**	**32.5±3.4**	**36.7±4.4**	**33.2±4.9**	**34.5±2.5**
p	0.487		0.032*		0.107		0.581	

**Sample No.**	**Sample 1**		**Sample 2**		**Sample 3**		**Sample 4**	
**Marker/ Study**	**o-Tyr/B**	**o-Tyr/C**	**o-Tyr/B**	**o-Tyr/C**	**o-Tyr/B**	**o-Tyr/C**	**o-Tyr/B**	**o-Tyr/C**
**Female**	137	122	176	176	126	146	133	144
**Male**	164	135	157	165	162	152	125	134
**Male**	158	134	172	179	154	124	148	137
**Male**	132	141	164	184	167	133	139	158
**Female**	145	128	184	188	145	132	146	119
**Female**	165	126	174	183	130	152	138	139
**Mean±SD**	**150±15**	**131±7.3**	**171.2±9.9**	**179.2±8.5**	**147±18**	**140±12**	**138.2±8.9**	**139±13**
p	0.022*		0.181		0.432		0.962	

**Sample No.**	**Sample 1**		**Sample 2**		**Sample 3**		**Sample 4**	
**Marker/ Study**	**3-ClTyr/B**	**3-ClTyr/C**	**3-ClTyr/B**	**3-ClTyr/C**	**3-ClTyr/B**	**3-ClTyr/C**	**3-ClTyr/B**	**3-ClTyr/C**
**Female**	73	75	78	79	74	71	74	70
**Male**	75	78	79	83	72	67	76	72
**Male**	72	74	84	81	77	69	69	73
**Male**	78	73	76	75	74	73	73	75
**Female**	71	69	76	79	71	72	75	74
**Female**	73	65	80	81	75	72	77	73

**Mean±SD**	**73.7±2.6**	**72.3±4.9**	**78.8±3.1**	**79.7±2.9**	**73.8±2.2**	**70.7±2.4**	**74±3**	**72.8±1.8**
p	0.584		0.656		0.046*		0.449	

**Sample No.**	**Sample 1**		**Sample 2**		**Sample 3**		**Sample 4**	
**Marker/ Study**	**3-NOTyr/B**	**3-NOTyr/C**	**3-NOTyr/B**	**3-NOTyr/C**	**3-NOTyr/B**	**3-NOTyr/C**	**3-NOTyr/B**	**3-NOTyr/C**
**Female**	102	98	132	124	115	87	94	84
**Male**	89	94	131	130	92	92	101	85
**Male**	112	99	128	128	94	94	113	105
**Male**	106	82	120	115	98	88	93	93
**Female**	99	84	125	126	121	76	105	86
**Female**	111	87	116	122	88	91	97	102
**Mean±SD**	**103.2±9**	**90.7±7.7**	**125.3±6.6**	**124.2±5.6**	**101±14**	**88±6.7**	**100.5±8**	**92.5±9.6**
p	0.032*		0.758		0.073		0.163	

**Sample No.**	**Sample 1**		**Sample 2**		**Sample 3**		**Sample 4**	
**Marker/ Study**	**8-OHdG/B**	**8-OHdG/C**	**8-OHdG/B**	**8-OHdG /C**	**8-OHdG/B**	**8-OHdG/C**	**8-OHdG/B**	**8-OHdG/C**
**Female**	86	76	90	103	87	85	85	84
**Male**	75	87	106	98	81	76	92	82
**Male**	74	89	89	105	90	79	86	89
**Male**	81	77	108	92	91	74	87	93
**Female**	88	80	98	99	94	80	89	84
**Female**	83	91	99	111	76	88	84	85
**Mean±SD**	**81.2±9**	**83.3±6.8**	**98.3±8.3**	**101.3±6.9**	**86.5±7.1**	**80.3±5.6**	**87.2±3.1**	**86.2±4.3**
p	0.587		0.527		0.140		0.665	

**Sample No.**	**Sample 1**		**Sample 2**		**Sample 3**		**Sample 4**	
**Marker/ Study**	**8-OHG/B**	**8-OHG/C**	**8-OHG/B**	**8-OHG /C**	**8-OHG/B**	**8-OHG/C**	**8-OHG/B**	**8-OHG/C**
**Female**	72	77	98	102	76	80	85	76
**Male**	77	72	96	94	92	78	83	71
**Male**	88	74	88	97	90	82	83	79
**Male**	83	76	105	98	85	86	88	84
**Female**	78	74	85	93	72	74	87	76
**Female**	81	79	101	97	94	75	81	72
**Mean±SD**	**79.8±5.8**	**75.3±2.6**	**95.5±8**	**96.8±3.3**	**84.8±9.4**	**79.2±4.7**	**84.5±2.8**	**76.3±5**
p	0.126		0.730		0.236		0.007**	

**Sample No.**	**Sample 1**		**Sample 2**		**Sample 3**		**Sample 4**	
**Marker/ Study**	**5-OHMeU/B**	**5-OHMeU/C**	**5-HMeU/B**	**5-OHMeU/C**	**5-OHMeU/B**	**5-OHMeU/C**	**5-HMeU/B**	**5-OHMeU/C**
**Female**	52	56	65	74	54	66	54	57
**Male**	52	58	78	77	46	55	56	52
**Male**	58	63	76	78	60	56	53	53
**Male**	58	65	82	80	62	54	44	55
**Female**	55	66	69	73	56	51	51	55
**Female**	48	65	75	77	48	61	48	54
**Mean±SD**	**53.8±4.1**	**62.6±4.4**	**74.2±6.5**	**76.5±2.7**	**54.3±6.7**	**57.2±5.7**	**51±4.6**	**54.3±1.8**
p	0.009**		0.462		0.466		0.158	

**Sample No.**	**Sample 1**		**Sample 2**		**Sample 3**		**Sample 4**	
**Marker/ Study**	**LTB4/B**	**TB4/C**	**LTB4/B**	**LTB4/C**	**LTB4/B**	**LTB4/C**	**LTB4/B**	**LTB4/C**
**Female**	26	32	34	34	28	24	26	26
**Male**	31	30	38	44	31	29	25	28
**Male**	31	25	41	36	24	28	27	27
**Male**	28	23	36	34	26	22	27	29
**Female**	22	25	44	42	23	33	28	21
**Female**	19	26	30	33	28	30	18	28
**Mean±SD**	**26.2±5.1**	**26.8±3.6**	**37.2±5.2**	**37.2±4.9**	**26.7±3.1**	**27.7±4.2**	25.5±3.8	**26.5±3**
p	0.808		1.000		0.664		0.536	

**Sample No.**	**Sample 1**		**Sample 2**		**Sample 3**		**Sample 4**	

Marker/ Study	LTC4/B	LTC4/C	LTC4/B	LTC4/C	LTC4/B	LTC4/C	LTC4/B	LTC4/C
**Female**	59	60	79	88	66	54	67	52
**Male**	79	63	84	92	65	51	56	66
**Male**	56	57	88	94	64	55	62	61
**Male**	68	55	78	91	68	58	76	55
**Female**	65	59	83	85	61	54	80	58
**Female**	68	62	95	85	63	63	62	55
**Mean±SD**	**65.8±8.5**	**59.3±3.2**	**84.5±6.6**	**89.2±3.9**	**64.5±2.5**	**55.8±4.4**	**67.2±9.6**	**57.8±5.3**
p	0.136		0.185		0.002**		0.074	

Sample No.	Sample 1		Sample 2		Sample 3		Sample 4	
**Marker/ Study**	**LTD4/B**	**LTD4/C**	**LTD4/B**	**LTD4/C**	**LTD4/B**	**LTD4/C**	**LTD4/B**	**LTD4/C**
**Female**	25	23	32	30	30	22	24	23
**Male**	21	25	29	33	25	26	21	19
**Male**	21	20	37	35	28	20	27	25
**Male**	28	21	30	33	24	25	26	20
**Female**	22	25	35	37	26	26	28	21
**Female**	22	22	38	32	22	21	28	24
**Mean±SD**	**23.2±2.9**	**22.7±2.2**	**33.5±3.9**	**33.3±2.5**	**25.8±3**	**23.3±2.8**	**25.7±2.9**	**22±2.5**
p	0.754		0.935		0.183		0.047*	

Sample No.	Sample 1		Sample 2		Sample 3		Sample 4	
**Marker/ Study**	**LTE4/B**	**LTE4/C**	**LTE4/B**	**LTE4/C**	**LTE4/B**	**LTE4/C**	**LTE4/B**	**LTE4/C**
**Female**	36	43	44	56	47	33	39	38
**Male**	51	45	52	55	44	41	32	38
**Male**	40	40	50	52	38	39	46	29
**Male**	45	35	53	50	43	37	34	30
**Female**	41	38	54	48	41	40	42	33
**Female**	46	33	58	51	38	36	40	37
**Mean±SD**	**43.2±5.5**	**39±4.8**	**51.8±4.9**	**52±3.2**	**41.8±3.7**	**37.7±3.1**	**38.8±5.4**	**34.2±4.3**
p	0.213		0.948		0.071		0.143	
